# Editorial

**Published:** 1842-01-01

**Authors:** 


### THE MEDICAL EXAMINER.

### PHILADELPHIA, JANUARY 1, 1842.

A new series of the Medical Examiner is this day commenced, with considerable modifications of form, price, and editorial arrangements. Our readers of the past year have been made acquainted with the character of the changes which have been for some time contemplated in the journal, and which now take effect ; but, as the new series begins with a considerable accession of subscribers, and with the prospect of many more, it is proper that we should present a brief outline of the present editorial organization of the journal, the character which we shall aim to impress upon it, and the principles by which its course will be directed. Drs. BIDDLE and GERHARD will continue to be co-editors of the new series, with particular supervision of the departments which they have conducted under the old arrangements. Dr. REYNELL COATES becomes acting editor of the journal, and will moreover specially take charge of the departments of surgical pathology and therapeutics. Aided by a large circle of scientific friends, and, with the resources and connections of this medical metropolis at command, the editors confidently believe that they will be able to make the Examiner as valuable as any similar publication. It is, and will ever continue to be free from local or party bias. Advocating the interests of the entire profession, it will dwell rather on principles than on men, never losing sight of a main object—the advancement of the art of healing, and the elevation of the medical character.

It forms no part of our design to publish long and elaborate articles, which are better fitted to appear as separate treatises in the ponderous quarterlies, nor do we intend to charge our pages with the controversial discussions—interesting only to the parties concerned—which find their way into some of our cotemporaries. Our object is to publish a journal devoted to the dissemination of useful information and sound principles of medical politics and ethics, of a form and size that will render it a convenient *vade mecum* to the busy practitioner, as well as a compendium of valuable matter for future reference and study. We shall endeavour to make the journal a reflection of the current medical literature of the day, allotting, always, ample space to selections from the American and European periodicals. These selections will in general be annotated, and will often assume the character of critical bibliography, rather than of mere transfer of the matter of other journals. Notices and reviews of the new publications will constantly appear.

In the clinical department we shall procure well digested reports from the different hospitals, and, not unfrequently the editors themselves will be the reporters. This will be the case, also, in the reports of proceedings of societies ; and the history and polity of such medical institutions, here and elsewhere, will be considered as proper subjects of comment whenever the broad interests of the profession appear to render such comment necessary.

In the department of original communications, we have received promises of assistance from many able friends, and shall always be happy to give the earliest publicity to whatever is really valuable in the favours of our correspondents; but, throughout every part of the journal, it will be our first wish to consider the practical value of the matter as paramount to mere variety of origin, the editors being desirous of building the reputation of the Examiner upon facts rather than names—things rather than words.

At that season of the year in which the annual influx of medical students to the school of Philadelphia is about to commence, we shall endeavour to inform preceptors and pupils at a distance of the nature and extent of the arrangements for instruction during the coming medical session.

Among the subjects of occasional discussion in our pages, will be the laws of medical etiquette, the management of consultations, the relations of physicians with the public, with courts of justice, and with individuals in other professions.

---

Owing to the peculiar character of other engagements, Dr. W. Poyntell Johnston relinquishes his editorial connection with the Examiner. The journal will, however, continue to have the benefit of his valuable assistance as an active collaborator.

